# Systematic Review: Recommendations of Levels of Physical Activity among Colorectal Cancer Patients (2010–2019)

**DOI:** 10.3390/ijerph18062896

**Published:** 2021-03-12

**Authors:** Jineui Hong, Jungmin Park

**Affiliations:** School of Nursing, Hanyang University, Seoul 04763, Korea; honghong@hanyang.ac.kr

**Keywords:** cancer, colon, rectum, colorectal cancer, physical activity, recommendation, prognosis, mortality, recurrence

## Abstract

Background: It is necessary to engage in an appropriate level of physical activity to improve the prognoses of colorectal cancer patients, yet no guidelines currently exist. The goals of this systematic review are to determine the impact of levels of physical activity on the prognoses of colorectal cancer patients and to suggest recommended guidelines for levels of physical activity. Methods: This systematic review was conducted along PRISMA guidelines. Per the inclusion criteria, papers published in academic journals in English from 2010 to 2019 were selected. A literature search was performed on PubMed (Medline), and the results of the selected studies were qualitatively synthesized. Results: Of the 13 cohort studies included in this systematic review, most studies were conducted in the United States (N = 7). Immobility or low levels of physical activity adversely affected the prognoses of colorectal cancer patients. Contrarily, high levels of physical activity increased the survival rate in people with colorectal cancer. Conclusion: For colorectal cancer patients, a level of physical activity of 17.5 to 35 MET hours per week is strongly recommended, which has been shown to reduce mortality by approximately 30 to 40%. Patients with limited physical capacity should maintain a minimum level of physical activity (≥3.5 MET hours/week).

## 1. Introduction

In 2018, an estimated 1.8 million cases of colorectal cancer were recorded worldwide, the third highest incidence (10.2%) of all carcinomas [1]. In addition, colorectal cancer is the second most common reason for death (9.2%) among cancer patients, accounting for 861,663 recorded deaths [1]. The United States Preventive Services Task Force (USPSTF) strongly recommends determining colorectal cancer diagnosis via the use of endoscopies, fecal examinations, and CT scans, given that polyps in the pre-cancerous stage are removed with an endoscope, allowing for the incidence of colorectal cancer to be reduced; doing so can reduce the mortality rate of colorectal cancer through early detection [2]. In 2018, 68.8% of Americans aged between 50 and 75 were screened for colorectal cancer [3]. Nevertheless, the ratio of colorectal cancer patients under 55 years old doubled across all age groups over a 20-year span, and accounts for about one-third of the total. This results in the need for additional efforts in reducing the incidence and promoting the early detection of colorectal cancer [4]. Due to these skyrocketing rates of colorectal cancer, deaths from this affliction will increase by an estimated 60.0–71.5% by 2035 (compared to 2016) [5].

Physical activity has a positive effect on the prognosis of colorectal cancer patients [6]. For active patients, the risk of colorectal cancer decreases by about 15% [7]. Physical activity is defined as the physical movements produced by skeletal muscles that result in energy expenditure [8]. Common physical activities consist of leisure activities, home activities, occupational activities, and mobile activities; the level of physical activity can be evaluated by calculating its type and duration [9]. Moreover, physical activity is a factor that can benefit one’s health through individual efforts [10].

The level of physical activity is evaluated by the metabolic equivalent task (MET) method; the MET score is then multiplied by the rate of energy expended during various physical activities, according to the baseline rate of energy expended at rest [11]. The criteria for high levels of physical activity vary depending on the researcher, and are suggested to range from 17.5 MET hours/week (hour/week) to 56 MET hours/week, depending on the study [12,13,14]. It has further been found that high levels of physical activity are associated with a 13 to 16% reduction of one’s risk of developing colorectal cancer [15]. High levels of physical activity can not only reduce the risk of developing colorectal cancer [7], but can also prevent various chronic diseases [16]. One study showed that, even in patients already diagnosed with colorectal cancer, the group engaging in high levels of physical activity had an improved prognosis, with a lower mortality rate than the group engaging in low levels of physical activity [17]. High levels of physical activity (35.5 MET hours/week, approximately 60–75 min/day of moderate-intensity exercise) have been recommended, because such activity has proven to prevent the increase in all-cause mortality when compared to the inactive group [18]. In particular, high levels of physical activity were effective in increasing the survival probability of colorectal cancer patients by 40% [17]. The physically active group had a lower recurrence rate than the inactive group, which is another representative prognostic indicator; however, the recommended activity level is not discussed [19]. The reason for this recommendation is that physical activity was important to cancer patients, and the American Cancer Society recommended moderate or active activity for all cancer patients through its Physical Activity Guidelines [20].

As such, related studies have examined the relationship between the level of physical activity and the incidence of colorectal cancer, or the effect levels of physical activity have on the mortality rate of colorectal cancer patients [15,18]. To improve the prognosis of colorectal cancer patients, physical activity is required [17]. Despite this, there are currently no guidelines for the level of physical activity recommended for such patients. Throughout our research, we derive the recommended level of physical activity for colorectal cancer patients. Thus, this systematic review intends to evaluate the effect that levels of physical activity have on one’s colorectal cancer prognosis, along with the appropriate recommended level of exercise for these patients. This study further aims to understand the concept of levels of physical activity, including physical activity as well as its intensity and frequency. Importantly, this study’s purpose is to identify the effects of levels of physical activity on the prognosis of colorectal cancer patients and to present basic data on the recommended amount of physical activity.

## 2. Materials and Methods

### 2.1. Inclusion and Exclusion Criteria

The relevant inclusion and exclusion criteria that were based on the population, intervention, comparison, outcome, and study design (PICOS) and guided this study were as follows: (1) population: colorectal cancer patients had to be over 18 years of age; (2) intervention (exposure), comparison, outcomes: evaluation of the levels of physical activity and patient prognosis (death and recurrence) had to be present; (3) study design: cohort study (causal relationship could be confirmed); (4) research needed to be written in English; (5) the papers needed to be submitted to academic peer-reviewed journals within the last 10 years (to increase the credibility of our findings and to keep them up to date); and (6) the papers could not cover reviews or meta-analyses.

### 2.2. Searching and Screening

**Figure 1:**
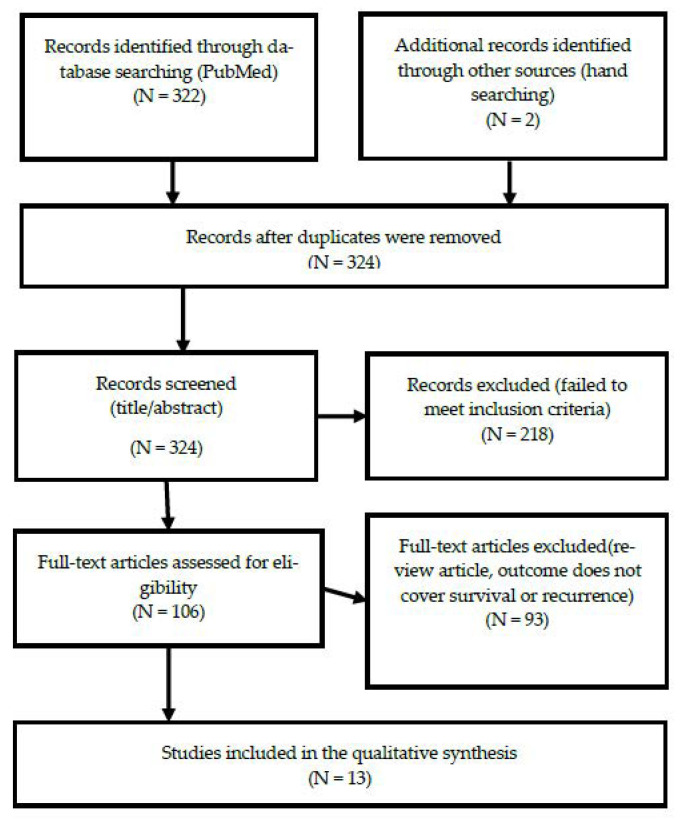
Flow diagram.

For this study, a systematic review was conducted according to PRISMA guidelines (see Figure 1). According to the inclusion criteria, a literature search was conducted in the PubMed database; related papers were then searched for by hand (via the references of the searched for and selected papers) and checked. The main keywords used in the search were generated using the MeSH Keyword generator and tree. The MeSH keyword generator selected “Exercise,” “Colorectal Neoplasms,” and “Colonic Neoplasms” as appropriate keywords. As a result of checking the MeSH tree, intestinal neoplasms emerged as the top keyword, encompassing colonic neoplasms and colorectal neoplasms. Finally, we searched for “(exercise [MeSH Terms]) AND (intestinal neoplasms [MeSH Terms]).” Through the search process, a filter was used to determine the publication year (from January 2010 to December 2019) to encompass the last 10 years. In total, 322 papers were found within the literature database. We reviewed the papers selected through search and tried to find additional papers suitable for our systematic review by checking the references written in the papers, and finally, we added two papers through manual searching. These papers were managed using the bibliographic management program EndNote. There were no duplicate documents, and, as such, the first selection process retrieved 324 papers.

In the first screening process, the title and abstract were checked according to the study’s inclusion and exclusion criteria. This resulted in the exclusion of 218 papers, leaving 106 papers. The first screening included papers published in academic journals, research on colorectal cancer patients, papers related to physical activity and the prognosis of the subjects, and papers published over the last 10 years (from January 2010 to December 2019). The first screening process was completed by excluding papers that either did not meet the inclusion criteria of being written in English or did not have abstracts.

Full-text access was confirmed via a secondary screening process, resulting in the exclusion of an additional two papers for which no full text existed, and 16 papers that were either reviews or meta-analyses. Thirteen papers were finally selected, excluding the remaining 74 studies, as they did not evaluate levels of physical activity in patients with colorectal cancer, or did not provide a prognosis. One study was excluded because it was a cross-sectional study, and the causal relationship could not be confirmed. The document collection and data extraction process was reviewed and finally agreed upon by two researchers through several meetings, and there were no disagreements.

### 2.3. Assessment of Methodological Quality

**Figure 2:**
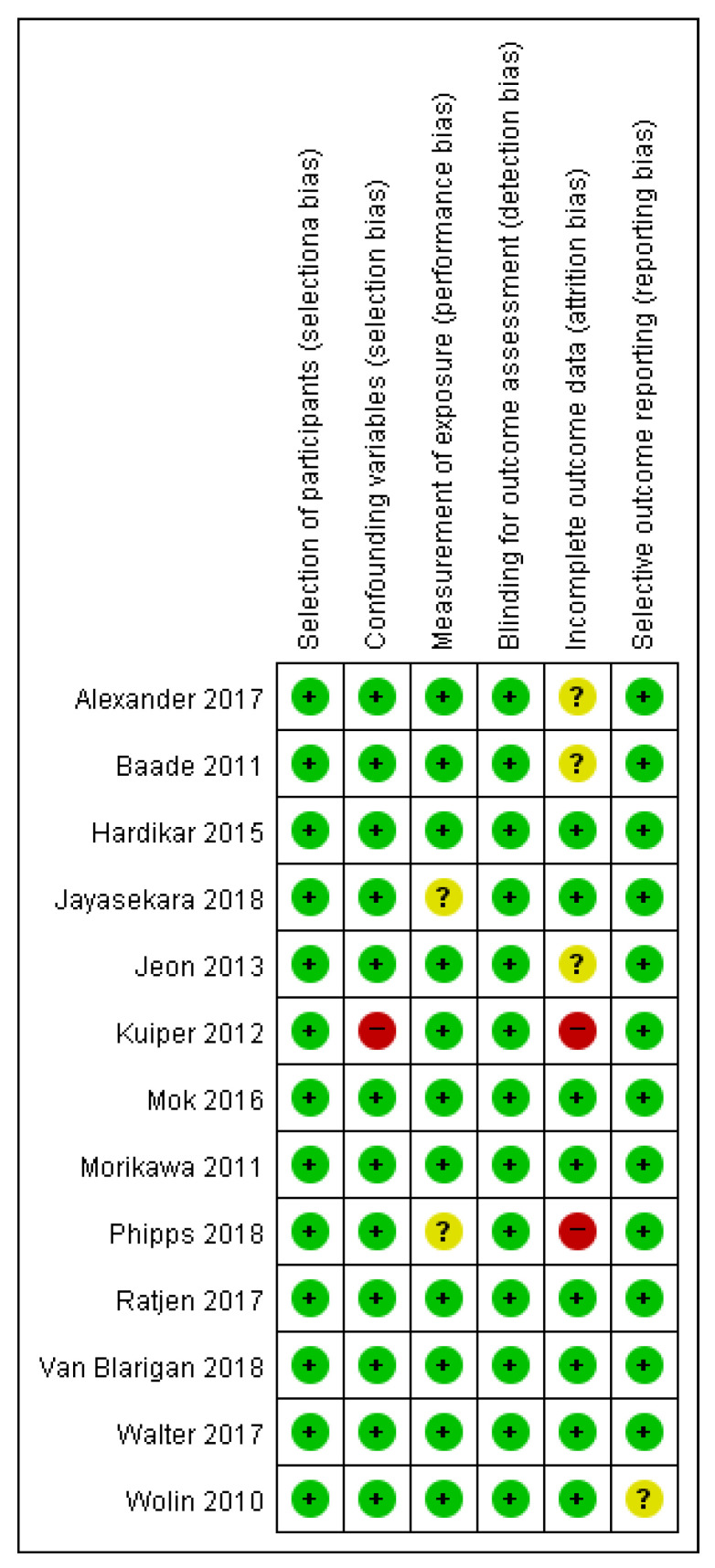
Summarized results of the quality evaluation for literature using RoBANS.

This study used the risk of bias assessment tool for non-randomized studies (RoBANS) [21]. This tool was developed in 2009 to evaluate the quality of non-random research, and its validity was verified. It is a form that combines the checklist method and the domain evaluation method. It is used to evaluate the quality of non-random assignment comparative clinical trial studies, cohort studies, patient–control studies, and post-war studies. It consists of six evaluation areas: comparability of the target group, selection of the target group, confounding variable, exposure measurement, evaluator blinding, result evaluation, incomplete result data, and selective result report. In the RoBANS tool, the risk of bias in each evaluation area was evaluated as low risk, unclear risk, or high risk, and two researchers independently performed it. Inconsistent items among evaluators reached consensus through discussion. Figures for the quality assessment results were obtained using Cochran’s revman version 5.4 (see Figure 2).

### 2.4. Data Extraction

The focus of data analysis was to identify recommendations of levels of physical activity among colorectal cancer patients through the literature review. Selected papers were analyzed along a process of (1) data reduction, (2) data display, (3) data comparison, and (4) the drawing of conclusions [22]. Papers on the relationship between the level of physical activity and prognosis, such as recurrence and death or survival, were summarized and analyzed according to author, purpose, setting, design, data, duration, physical activity, and outcomes (see Table 1). After confirming the general characteristics of the studies of the selected papers, the criteria and methods for evaluating the levels of physical activity of each study were identified and analyzed. The effects of each study on disease-free survival, disease-specific mortality, disease-specific survival, overall survival, and all-cause mortality were summarized according to the level of physical activity in colorectal cancer patients. In other words, a qualitative synthesis was conducted to see how the level of physical activity affected the prognosis. Finally, the conclusion was drawn by analyzing the recommended level of physical activity to improve the prognosis of colorectal cancer patients based on the qualitatively synthesized data.

## 3. Results

### 3.1. General Characteristics of the Studies

Among the papers related to physical activity and prognosis of colorectal cancer patients, the researchers finally selected 13 papers that met the specific inclusion criteria. The sample sizes of the studies varied from 181 [23] to 226,089 patients [12]; the average sample size was 12,462 patients. The research design of the selected papers did not include experimental studies; all papers were observational studies. A total of 13 studies were cohort studies, with 11 being prospective cohort studies [6,13,14,17,24,25,26,27,28,29,30] and two retrospective cohort studies [12,23]. The countries and population groups in which the selected papers were conducted consisted of: seven articles concerning the U.S. [13,17,24,26,27,29,30]; two articles concerning Germany [6,14]; two articles concerning Australia [25,28]; one article concerning Korea [12]; and one article concerning the UK [23]. Three studies evaluated levels of physical activity before the diagnosis of colorectal cancer [13,23,28], while 12 studies evaluated levels of physical activity after diagnosis [6,12,14,24,25,26,27,29,30,31]. One study compared the results from before and after diagnosis [17].

### 3.2. Levels of Physical Activity

The most widely-used physical activity variable was the metabolic equivalent task (MET) [6,12,13,14,17,24,26,27,29]. Researchers in these studies obtained the MET score by multiplying the rate of energy expended during various physical activities against the baseline resting metabolic rate [11]. The MET value is multiplied by the activity duration and converted into a total weekly MET value [11]. Studies using MET did not use the continuous variable calculated as raw MET hours/week, resulting in the continuous variable being binned [6,12,13,14,17,24,26,27,29]. The MET scores for each major physical activity are: 3.0 for walking; 6.0 for cycling; 6.0 for sports; 4.0 for gardening; 3.0 for housework; and 4.5 for home repairs [6]. The basic MET score is 1.0, which occurs when sitting and resting [6,11]. In addition, researchers used a method of categorizing light, medium-intensive, or high-intensive activities based on the intensity thereof [13]. The light activities (slow dancing, bowling, and golf) received an MET score of 3.0; the medium-intensive activities (biking, exercise machines, calisthenics, easy swimming, dancing) were scored as a value of 4.0 MET; and the highly intensive activities (aerobics, jogging, tennis, and swimming laps) were considered to score a value of 7.0 MET [13].

The reference points for low and high levels of physical activity were different for each study, but seven papers considered variables within the 17.5–18 MET score range as an important criteria range [12,13,17,24,26,27,29]. Two studies compared MET scores by dividing them into quartiles [6,14]. There have been studies that considered weekly MET hours of 0.0 as little or no physical activity and fewer than 3.0 weekly MET hours as very poor or low physical activity [12,13,17,27]. Studies have defined weekly MET hours above 35.0 as the highest level of physical activity [13].

Phipps’ study, which did not use MET scores, showed how often moderate-intensity physical activities (golf, garden care, long walks, bowling, etc.) and high-intensity physical activities (jogging, racquet sports, swimming, aerobics, etc.) were performed during leisure time [30]. Depending on the intensity, the group was divided into four groups, ranging from the non-physical group to the high physical activity group [30]. Jayasekara’s study scored physical activities, such as exercise and walking, before the patients’ diagnosis and used them as variables for further analysis [28]. Alexander’s study evaluated physical activity in terms of the patient’s ability to climb stairs [23]. Mok’s study used MET values, but duration and frequency also served as variables of physical activity [12]. Baade’s study calculated the level of physical activity with walking time within one week and moderate or intense physical activity time (according to the Australian Physical Activity Guideline), and divided the results into the inactive group, the insufficiently active group, and the sufficiently active group [25]. Baade also investigated the changes in physical activity at 12 months [25]. Latizen’s study considered the TV viewing time using periods of less and more than 2 h, as well as less and more than 4 h and sitting time as a variable. MET scores were used as a supplementary measure [6].

### 3.3. The Effect of Levels of Physical Activity on the Prognosis of Colorectal Cancer Patients

The prognosis of colorectal cancer patients (according to the degree of physical activity they participated in) consisted of: (1) the death and survival of colorectal cancer patients; and (2) the recurrence of colorectal cancer. Studies concerning the mortality and survival of colorectal cancer patients as outcomes compared disease-specific mortality or disease-specific survival along the lines of levels of physical activity [12,13,14,17,24,25,26,28,29,30]; all-cause mortality and overall survival were also compared within this framework [6,13,14,17,23,25,27,28,29,30]. Studies that included recurrence as a prognostic index were compared with either the hazard ratio [14,29,30] after setting recurrence or disease-free survival as the baseline prognostic value [14,29,30].

#### 3.3.1. Recurrence and Disease-Free Survival

The high level of physical activity, as suggested in some studies, ranged between 29.2–56.2 MET hours/week, and improved the disease-free survival rate (HR: 0.78, 95%; CI: 0.65–0.93) of patients without metastasis in the high-level physical activity group [14]. This high level of physical activity applied to those who jogged for about 5–8 h a week, and the jogging value was 7.0 MET hours/week [13]. In addition, when physical activity was performed more than twice a month, the disease-free survival rate improved (HR: 0.82, 95%; CI: 0.69–0.99) compared to those who exercised less than twice a month [30]. High levels of physical activity among non-smokers, T3 (cancer stage) patients, and obese individuals had a high effect on preventing the recurrence of colorectal cancer [30].

#### 3.3.2. Disease-Specific Mortality

In observing the results of comparing the disease-specific mortality based on levels of physical activity, it was observed that it is possible to reduce the disease-specific mortality rate of colorectal cancer when levels of physical activity were maintained over 17.5 MET hours/week (HR: 0.81, 95%; CI: 0.66–0.99 or HR: 0.64, 95%; CI: 0.45–0.91) [12,24]. Regardless of before or after diagnosis, a level of physical activity above 18.0 MET hours/week is effective in reducing mortality; however, the level of physical activity after diagnosis (HR: 0.29, 95%; CI: 0.11–0.77) was more effective than the one prior to diagnosis (HR: 0.68, 95%; CI: 0.41–1.13) [13]. Increasing the level of physical activity by less than two hours per week (HR: 0.68, 95%; CI: 0.48–0.97) or more than two hours per week (HR: 0.64, 95%; CI: 0.44–0.93) proved effective in lowering disease-specific mortality rates [25]. More specifically, a difference appeared in the risk of death according to men’s regular physical activity (HR: 0.75, 95%; CI: 0.58–0.967) [12] and physical activity in stage II tumors (HR: 0.25, 95%; CI: 0.10–0.60) [28]. Pathologically, even in patients with negative nuclear CTNNB1, high levels of physical activity (HR: 0.33, 95%; CI: 0.13–0.81) produced a mortality reduction effect [26].

#### 3.3.3. Disease-Specific Survival

In a study comparing disease-specific survival, a high level of physical activity ranging between 29.2 and 56.2 MET hours/week increased disease-specific survival (HR: 0.66, 95%; CI: 0.52–0.83), which also increased in the group above 35 MET hours/week (HR: 0.70, 95%; CI: 0.52–0.96) [14,17]. Moreover, even if levels of physical activity were not that high, a modest amount of physical activity of 3.5 MET hours/week or more (HR: 0.58, 95%; CI: 0.0.39–0.72) helped increase the survival rate [17].

#### 3.3.4. Overall Survival

The overall survival rate for 8.75–17.4 MET hours/week was higher (HR: 0.64, 95%; CI: 0.45–0.92) than for levels of physical activity of 0.0 or less than 8.75 MET hours/week, and higher than 17.5 MET hours/week (HR: 0.58, 95%; CI: 0.42–0.81) [29]. It has been shown that the survival rate increases as the level of physical activity increases [29]. In addition, survival rates improved even when the patient engaged in the minimum level of physical activity of 3.5 MET hours/week (HR: 0.53, 95%; CI: 0.39–0.72) [17] or when moderate-intensity exercises (HR: 0.76, 95%; CI: 0.63–0.93) were performed more than twice a month [30].

#### 3.3.5. All-Cause Mortality

Comparing all-cause mortality, the physically active group had a lower risk of mortality (HR: 0.53, 95%; CI: 0.36–0.80) than the less active group [6]. Even with insufficient levels of physical activity (which are greater than 0.0), the risk of death was lower than that of not doing any physical activity (HR: 0.72, 95%; CI: 0.57–0.91). The risk of death decreased by 31% as a result of increasing the physical activity time of patients diagnosed with colorectal cancer by more than 2 h [25]. At the point of measurement, high levels of physical activity proved to be effective in reducing mortality regardless of when the physical activity was engaged in (before or after diagnosis) [13]. Nonetheless, patients with more than 18.0 MET hours/week of activity after receiving their diagnosis (HR: 0.41, 95%; CI: 0.21–0.81, *p* trend = 0.005) had a greater reduction in mortality than those with the same amount of activity before being diagnosed (HR: 0.63, 95%; CI: 0.42–0.96) [13]. One study evaluated the patients’ ability to climb stairs, indicating that the risk of death increased in patients who could not climb stairs without resting (HR: 3.31, 95%; CI: 1.13–9.66) than those who were able to do it without resting [23]. The risk of mortality was reduced when sports (HR: 20, 95%; CI: 0.20–0.59), walking (HR: 0.65, 95%; CI: 0.43–1.00), and gardening activities (HR: 0.62, 95%; CI: 0.42–0.91) of 20.0 MET hours/week or higher were engaged in [6]. In addition, watching TV for more than 4 h per week resulted in a higher rate of mortality (HR: 1.45, 95%; CI: 1.02–2.06) than watching TV for less than 2 h a week [6]. Even among patients with a recurrence of colorectal cancer, high levels of physical activity contributed to a 29% reduction in mortality risk [27].

### 3.4. Recommended Level of Physical Activity to Improve the Prognosis of Colorectal Cancer Patients

This study was able to corroborate that high levels of physical activity in patients with colorectal cancer had a good effect on their prognoses. In general, the higher the levels of physical activity, the better the prognosis [29]. In addition, it was found that even performing a low level of physical activity had a positive effect on patients’ prognoses compared to those not engaged in any physical activity [17]. In particular, a strong prognostic effect was observed in patients who engaged in physical activity levels above 17.5–18.0 MET hours/week [12,24]. Some studies found that very high physical activity levels have a greater effect; as such, if the patient’s physical ability level was sufficient, or if the patient liked to exercise, a weekly physical activity of 35.0 MET hours/week or more could be recommended [14,17]. Since prolonged immobility, such as that experienced when one’s TV viewing time increases, adversely affects the prognosis, it became desirable for people who had difficulties in performing physical activities to engage in at least minimal amounts of exercise [6]. Although it would not reach the optimal recommended level, physical activity itself would be an important factor in the prognosis, because even a minimum level of exercise could be effective over a state of immobility [17]. Through this systematic review, the recommended physical activity levels for colorectal cancer patients using MET scores could be classified into three categories. For patients with physical activity difficulties, level 1 (3.5 MET hours/week) or higher is recommended; for general patients, level 2 (17.5 MET hours/week) or higher is recommended; finally, for patients with excellent physical abilities, level 3 (35.0 MET hours/week) or higher should be recommended.

## 4. Discussion

As the number of survivors of cancer increases, such as colorectal cancer, interest in lifestyle and physical activity is becoming more important [32]. Physical activity, a kind of lifestyle behavior, can be improved through interventions [33]; as a result, physical activity is important because it can improve health via high levels of physical activity. One study of colorectal cancer patients indicated an advantage in their prognoses, whereby the survival rate of patients with high levels of physical activity was about 40% higher than that of patients with low levels of physical activity [17]. Additionally, previously-devised guidelines provided a general framework for all cancer patients, but since similar guidelines had not been produced specifically for colorectal cancer patients [20], it became necessary to suggest an appropriate level of physical activity. Through this systematic review, we have identified the concept of levels of physical activity and the amount of physical activity that can be recommended to improve the prognoses of patients with colorectal cancer.

Research concerning levels of physical activity needs to objectify the subjective response of physical activity; subsequently, it is important to present such research as a standardized value for comparison with other studies. Most of the literature that we reviewed to address this problem evaluated physical activity using the MET score [6,12,13,14,17,24,26,27,29]. The MET score is a method of calculating how much more one’s metabolism increases per physical activity, based on the resting metabolic rate—defined as a score of 1.0 [11]. We consider that the MET score method should be used in future studies, as it carries the advantage of easy synthesis of research results.

The physical activity ability of colorectal cancer patients can be assessed with the Eastern Cooperative Oncology Group (ECOG) performance status. The distribution of colorectal cancer patients’ physical activity ability is diverse: 59.8% (ECOG0) of the patients can live daily without physical activity restrictions; 25.7% (ECOG1) of the patients are capable of engaging in daily life and behavior, but are unable to perform heavy work; 10.4% (ECOG2) of the patients can engage in self-care but have a limited scope of ability in regard to physical activity; 3.6% (ECOG3) of the patients have severe physical activity restrictions; and 0.5% (ECOG4) of the patients are bedridden [34]. Since patients’ physical activity abilities are different, it was necessary to present the recommended level accordingly. In applying this framework, it is necessary to also consider the recommended levels of physical activity for those patients who are bedridden (ECOG 4) and those with severe restrictions to their abilities to perform physical activities (ECOG2-3) [35]. In assessing the results of the review, we recommend that even those patients who have difficulties in performing physical activities reduce the amount of time lying down and watching TV (taken as a proxy for sedentary behavior) [6]. It was desirable to educate the patient to slowly climb and descend nearby stairs [23] or to perform physical activity of at least 3.5 MET hours/week [17]. It is suggested that 17.5 MET hours/week, which is the most effective level for improving a patient’s prognosis, is recommended for general patients who are not physically restricted, and 35.0 MET hours/week is recommended for people with an excellent ability to perform physical activities or exercises. This result is meaningful in that it can provide guidelines regarding physical activity recommendations for patients with colorectal cancer.

The intensity of physical activity can be divided into three levels, according to the MET score: (1) light-intensity activities (< 3.0 MET); (2) moderate-intensity activities (3.0 to 6.0 MET); and (3) vigorous-intensity activities (> 6.0 MET) [36]. To meet the recommended levels suggested in this review, activity above level 1 (3.5 MET hours/week) equates to about 70 min of walking (about 3.0 MET) or about 35 min of cycling (about 6.0 MET); for level 2 (17.5 MET hours/week), about 350 min (or roughly 6 h) of walking or 175 min (or 3 h) of cycling; and level 3 patients (35.0 MET hours/week) must engage in about 700 min (12 h) of walking or 350 min (approximately 6 h) of cycling [11,36]. The ACS guidelines for all cancer patients recommend that they perform at least 150 min of moderate activity or 75 min of vigorous aerobic exercise per week [37]. When this activity is calculated via MET scores, it is approximately equal to 7.5–10.0 MET hours/week. This number of hours is lower than the level of physical activity for colorectal cancer patients recommended in this review, which is presumed to be because all cancer patients were targeted. In addition, the ACS guidelines suggested that one should do exercises requiring muscle strength for about two days every week; furthermore, even if one is diagnosed with cancer, it is recommended to prevent physical activity reduction and maintain a normal daily life [37]. Because the ACS guideline covers all cancer patients and does not reflect physical ability, this review is considered to play an important role, as it addressed and introduced guidelines for colorectal cancer patients. When providing recommendations, it will be helpful to the patient to provide a table of recommended activity hours for each physical activity in an easy-to-read manner.

As shown in this systematic review, the effect of high levels of physical activity is certain to lower recurrence and mortality rates, which are important prognoses of colorectal cancer. Furthermore, physical activity reduces insulin resistance, reduces inflammation, increases myokine secretion by the musculoskeletal system, and decreases colon transit time, each of which is believed to have an effect on reducing the carcinogenic process of colorectal cancer and other comorbidities [32]. Nonetheless, the molecular mechanism underlying the manner in which physical activity improves the prognosis of colorectal cancer patients remains unknown [32].

Not only do high levels of physical activity lower recurrence and death, the most important prognostic indicators related to life, but have other additional benefits. Physical activity is effective in alleviating depression [38] and improves the quantity and quality of one’s sleep [39]. Because it immunologically suppresses carcinomas, it is also helpful in treatment procedures [40] and, finally, improves one’s quality of life [41]. Physical activity serves as an exceedingly beneficial treatment because it has few side effects and is non-invasive, cost-effective, and accessible [38]. It is important to note, however, that 68% of colorectal cancer survivors were found to engage in little physical activity after receiving their diagnosis and undergoing treatment [42]. Factors that will reduce levels of physical activity nowadays are emerging [43]. Due to the spread of smartphones, along with their increased use, inactivity time has increased as well. Throughout the COVID-19 pandemic, average global physical activity dropped by between 5.5% and 27.3%; for instance, the more severe the pandemic was experienced in a locale, such as in Italy, the more the activity decreased (up to 47.8% decrease) [44]. Colorectal cancer patients are more likely to be affected, as their activity is being greatly reduced, which adversely affects their prognosis. Efforts to maintain the recommended levels of physical activity are, therefore, required.

Because a difference in physical activity ability for each patient exists, the recommended level of physical activity is estimated to be different. Even so, because previous research did not focus on or comprehensively analyze group characteristics, such as age and gender, there is insufficient evidence to tailor the level of physical activity. In future studies, certain problems might be solved via research using new measurement tools, such as wearable devices, or by analyzing sufficient amounts of big data along various variables. Wearable devices, including smart bands and belts, can easily, accurately, and abundantly obtain levels of physical activity, such as activity time and amount, by linking to a smartphone [45]. Using big data related to physical activity that has already been created enables researchers to present an accurate recommended level of physical activity for each patient. We propose an additional study using wearable devices or big data to suggest an appropriate level of physical activity for each subgroup, based on gender, age, and so on, for customized interventions.

Limitations

This systematic review has several limitations. First of all, despite the search being sufficient, using a single database is a weakness. Despite this, it was possible to select sufficient quality papers; by synthesizing the main results, it also became possible to propose a recommended level of physical activity for colorectal cancer patients. Another limitation is that the studies concerning the survival of cancer patients were significant in number, but, conversely, studies on recurrence were rare. Consequently, although the level of physical activity to reduce recurrence was not separately suggested, it could be a useful guideline for colorectal cancer patients because the studies approached the overall concept of prognosis, including both recurrence and mortality. Another limitation is that cancer research conducted in the United States, the British Commonwealth, or European countries was dominant. Only one paper had been written elsewhere, namely in South Korea, Asia [12], resulting in a limited ability to reflect regional and cultural characteristics. Since colorectal cancer is a global problem and increases rapidly with economic growth in developing countries [46], further research that can reflect the particular characteristics of various countries and regions is warranted.

## 5. Conclusions

This review of the literature on the prognosis of colorectal cancer patients according to their level of physical activity concludes the following. Immobility or low levels of physical activity adversely affect the prognoses of colorectal cancer patients. Conversely, since high levels of physical activity increase the survival rate of colorectal cancer patients and reduce the likelihood of death, physical activity should be actively encouraged for such patients to improve their prognoses. In general, as the level of physical activity increased, the prognosis tended to improve. Most of the results of the papers included in the review were 17.5 to 35 MET hours/week, which was the ideal level to improve the prognosis, which was found to reduce mortality by about 30 to 40%. Therefore, we strongly recommend level of physical activity level 17.5 to 35 MET hours/week for colorectal cancer patients. However, if the level of 17.5 MET hours/week or higher cannot be reached due to individual physical constraints, it is recommended to maintain a minimum level of physical activity (3.5 MET-hours/week) based on the results of some studies.

## Figures and Tables

**Table 1:** Summary of selected study characteristics, variables, and results.

First Author (Year)	Purpose, Setting, Design	Data & Characteristics	Variables	Outcomes	Key Findings
Wolin, K. Y. (2010)	**[Purpose]** Analyzing the risk of colorectal cancer incidence and mortality according to the level of physical activity among adults **[Setting]** The United States Population-based **[Design]** Prospective, cohort study	**[Data]** CPS II Nutrition Cohort (American Cancer Society) **[Duration]** Total: 1982–2006 PA: 1982, 1992, 1997 Cancer Dx. and mortality: 1998–2006 **[Cohort characteristics]** Total: 158,253 Incident CRC: 1863 Death d/t CRC: 846	**[Physical activity]** 1982: How much exercise do you get (work or play)? Low (none or slight) High (moderate or heavy) 1992: Cardiovascular exercise Walking, jogging, running, swimming, tennis, cycling, aerobics, dancing → MET < 17.5: low ≥ 17.5: high Increasing (low → high) Decreasing (high → low)	**[PA (level)—CRC]** - General None vs. 30 ≤ MET → HR: 0.72 (0.58–0.89) **[PA (level & change)—DSM]** Physical activity low (reference) Maintain high physical activity → HR: 0.81 (0.66–0.99) Decreased physical activity → HR: 0.91 (0.76–1.08) Increased physical activity → HR: 1.03 (0.74–1.45) (age-adjusted)	PA measurement after Dx (+) PA → DSM ↓ (+) PA → ACM ↓ (x) PA-Recur. ↓ (x) Compared to the low physical activity group, only the high physical activity maintenance group reduced their risk of colon cancer death
Baade, P. D. (2011)	**[Purpose]** Analyze the effect of physical activity and body mass index on the mortality rate of colorectal cancer patients **[Setting]** Queensland, Australia Population-based **[Design]** Prospective, cohort study	**[Duration]** Primary CRC patient Enrollment: 2003–2004 f/u: until 2008 (5 min, 12 min after Dx) **[Cohort characteristics]** Participants: 1825 Included CRC stage: 1, 2, 3 + unknown Excluded CRC stage 0, 4 (1.1%, 6.1%) mortality = 462 (25.3%) Mean dx. age: 67 (21–82)	**[Physical activity]** - The Active Australia Survey Sum of walking, moderate or vigorous PA time/overall 4 wks - PA level (Australia PA Guidelines) (1) sedentary: 0 min/wk (2) insufficiently active: 1–149 min/wk (3) sufficiently active: 150 ≤ min/wk → Kappa coefficient = 0.62 - PA change (five months, 12 months) (1) no change (2) increase 2 < h/wk (3) increase 2 ≤ h/wk	**[PA (level)—ACM]** 5 yrs mortality 25–28% ↓ **[PA (change)—DSM]** **PA↑ DSM 32~36% ↓** (1) no change (reference) (2) increase 2 < h/wk [HR: 0.68 (0.48–0.97)] (3) increase 2 ≤ h/wk [HR: 0.64 (0.44–0.93)] **[PA(change)—ACM]** **PA ↑ ASM 31% ↓** (1) sedentary (reference) (2) insufficiently active [HR: 0.72 (0.57–0.91)] (3) sufficiently active [HR: 0.69 (0.50–0.94)]	PA measurement after Dx (+) PA ↑ → DSM ↓ (+) PA ↑ → ACM ↓ (+) PA ↑ → Recur. ↓ (x)
Morikawa, T. (2011)	**[Purpose]** Determine the prognosis according to one’s body mass index (BMI) and post-diagnosis physical activity level in CTNNB1-activated colorectal cancer patients **[Setting]** The United States **[Design]** Prospective, cohort study	**[Data]** (1) The Nurses’ Health Study (since 1976) (2) The Health Professionals Follow-up Study (since 1986) **[Duration]** Until 30 June 2009 Treatment period: up until one year after diagnosis PA evaluation: one to four years after diagnosis **[Cohort characteristics]** CRC Stages: 1, 2, 3, 4, and 5 CRC patient: N = 955	**[Physical activity]** (1) Aerobic exercises walking (at their usual pace), jogging, running, cycling, swimming laps, playing racket sports (2) Lower-intensity exercise (yoga, toning, stretching) and other vigorous activities → MET scoring Reference: 18 MET h/wk	**[PA—DSM]** Nuclear CTNNB1 (−), stage (1, 2, 3) → adjusted HR: 0.33 (0.13–0.81) Nuclear CTNNB1 (+) → N/S	PA measurement after Dx (+) PA ↑ → DSM ↓ (+) PA ↑ → ACM ↓ (x) PA↑→ Recur.↓(x)
Jeon, J. (2013)	**[Purpose]** A study on the relationship between physical activity and survival period in patients with recurring colorectal cancer **[Setting]** The United States **[Design]** Prospective, cohort study	**[Data]** National Cancer Institute-sponsored Cancer and Leukemia Group B (CALGB) adjuvant therapy trial for stage III colon cancer **[Duration]** Enrollment: April 1999–May 2000 Stage 3 CRC pt. with recur. f/u until 9 November 2009. **[Cohort characteristics]** N = 237	**[Physical activity]** Total MET h/wk low: < 3 middle: < 3.0–17.9 high: ≥ 18	**[PA(Level)—ACM]** MET ≥ 18 Statistically significant difference (−) but mortality 29% ↓ [HR: 0.71 (0.46–1.11)] PA level ↑ → Mortality ↓ [trend *p* = 0.052]	PA measurement after Dx (+) → before recur. PA ↑ → DSM ↓ (x) PA ↑ → ACM ↓ (+) PA ↑ → Recur. ↓ (x) Physical activity affects the prognosis of recurrent patients.
Mok, Y. (2016)	**[Purpose]** Checking the correlation of CRC mortality according to the time and intensity of physical activity **[Setting]** South Korea Population-based **[Design]** Retrospective, cohort study	**[Data]** The Korean Metabolic Syndrome Mortality Study (KMSMS) 1994–2004 **[Duration]** f/u until 2014 **[Cohort characteristics]** N = 226,089 CRC death = 469	**[Physical activity]—Level** MET (MET h/wk) none, < 17.5, ≥ 17.5 - Duration (total h/wk) non, 2, 2–3, 4 - Frequency (frequency/wk): none, 1–3, 4 - Type: Jogging, jumping rope, walking, climbing, calisthenics, swimming, yoga, aerobics, or golf → Exercise * hour * frequency per week → MET h/wk	**[PA (level & change)—DSM]** - Overall 17.5 MET hours/week CRC mortality [HR: 0.64 (0.45–0.91)] - MEN Regular PA—CRC death [HR: 0.75 (0.58–0.97)]	PA measurement after Dx (+) PA ↑ → DSM ↓ (+) PA ↑ → ACM ↓ (x) PA ↑ → Recur. ↓ (x) Increasing total hours and intensity resulted in a decrease in colon cancer risk, but was not related to women.
Alexander, D. (2017)	**[Purpose]** Analyzing the relationship between modifiable behavioral factors and survival of CRC patients **[Setting]** The NHS Greater Glasgow and Clyde area, UK **[Design]** Retrospective, cohort study	**[Data]** Scottish Cancer Registry, National Scottish Death Records **[Duration]** Enrollment: 1 January 2012 to 31 December 2012 f/u until: 30 June 2015 **[Cohort characteristics]** N = 181 Total duration of F/U 480 person-years Mean age: Male = 68.7 (±9.2) Female = 67.0 (±9.9)	**[Physical activity]** The ability to climb stairs (1) Climbs stairs without stopping (2) Climbs stairs with stopping, cannot climb stairs	**[PA−ACM]** Able to climb stairs without resting: reference Unable to climb stairs without resting [HR: 3.31 (1.13–9.66)]	PA measurement before Dx (+) PA ↑ → DSM ↓ (x) PA ↑ → ACM ↓ (+) PA↑Recur.↓(x)
Ratjen, I. (2017)	**[Purpose]** Effects of physical activity, sleep, and TV viewing on all causes of death among CRC survivors **[Setting]** Regional cancer registry, Northern Germany **[Design]** Prospective, cohort study	**[Data]** Regional cancer registry (23 hospitals) **[Duration]** Dx: 1993–2005 F/u start: PA assessment date F/u end: date of death, last vital status assessment **[Cohort characteristics]** final total N = 1376 All-cause death N = 200 (14.5%) mean f/u = Seven years	**[Physical activity]** - MET walking: 3.0, cycling: 6.0, sports: 6.0, gardening: 4.0, housework: 3.0, home repair: 4.5, stair climbing: 8.0 × h/wk - Sports, cycling, gardening (MET h/wk) 0, > 0–10, > 10–20, > 20 - Housework, home repair, climbing stairs, walking: 0–10, > 10–20, > 20–30, > 30 - Duration of watching TV ≤ 2, > 2- < 4, ≥ 4 h/day - Total PA Q1 (0–64.5), Q2 (> 64.5–99.7), Q3 (> 99.7–144.9), Q4 (> 144.9)	**[PA−ACM]** PA Level Q4 (vs. PA Q1) [HR: 0.53(0.36–0.80)] sports > 20 = HR: 0.34 (0.20–0.59) walking > 20 = HR: 0.65 (0.43–1.00) gardening > 20 = HR: 0.62 (0.42–0.91) **[Watching TV−ACM]** ≤ 2 h/day = reference ≥ 4 h/day = HR: 1.45 (1.02–2.06)	PA measurement after Dx (+) → 6 yr survivors PA → DSM ↓ (x) PA → ACM ↓ (+) PA Recur. ↓ (x)
Walter, V. (2017)	**[Purpose]** To provide evidence for the relevance of physical activity before diagnosis of CRC on the prognosis after diagnosis **[Setting]** Southwest of Germany Population-based **[Design]** Prospective, cohort study	**[Data]** DACHS study (colorectal cancer: chances for prevention through screening)—population-based case-control study 2003~2010 CRC Dx. **[Cohort characteristics]** Over age 30 CRC Total: N = 3,121 Death: N = 868 CRC specific mortality = 635 Recurr. and meta = 623 mean f/u = 4.8 yrs	**[Physical activity]** Average physical activity (MET h/wk) Q1: 0.0–25.4 Q2: > 25.4–43.5 Q3: > 4.35–65.4 Q4: > 65.4 Recent physical activity (MET h/wk) Q1: 0.0–13.2 Q2: > 13.2–29.2 Q3: > 29.2–56.2 Q4: > 56.2 adjustment for occupational PA	**[Lifetime PA−Survival]** Overall Survival (−) CRC specific survival (−) Recurrence free survival (−) Disease-free survival (−) **[Latest PA−Survival]** PA ↑ Overall Survival ↑ (+) Q2 = HR: 0.81 (0.67–0.97) Q3 = HR: 0.64 (0.58–0.78), Q4 = HR: 0.75 (0.61–0.91) CRC specific survival (+) Q3 = HR: 0.66 (0.52–0.83) Disease-free Survival (+) Q3 = HR: 0.78 (0.65–0.93)	PA measurement after Dx (+) PA ↑ → DSM ↓ (+) PA ↑ → ACM ↓ (+) PA ↑ Recur. ↓ (−) Recent leisure activities have been associated with improved survival in non-metastatic CRC patients.
Jayasekara, H. (2018)	**[Purpose]** A study on the relationship between the lifestyle measured before diagnosis and the survival rate of CRC patients. **[Setting]** Melbourne, Australia **[Design]** Prospective, cohort study	**[Data]** Melbourne Collaborative Cohort Study Enrollment: 41,513 (1990–1994) **[Cohort characteristics]** Median age = 71 (44–87) CRC patient: N = 724 AJCC Excluding stage 4	**[Physical activity]** - Physical activity score 0, 0–3.9, 4–5.9, 6 or more - Exercise Non-exercisers: vigorous and moderate physical activity (never) exercisers: any regular exercise (≥ 1 time per week) - Walking (last 6 mths) for recreation or exercise: Not Walking: never/wk Walking: 1 ↑/wk	**[PA−Overall mortality]** **PA score (−)** PA ox (−) Walking (−) **[PA(level, exer.)−DSS/DSM]** **PA score (−)** PA ox (−) Walking (−) → subgroup stage II DSM PA ox (+) [HR: 0.25 (0.10–0.60)]	PA measurement before Dx (+) PA ↑ → DSM ↓ (+) in stage 2 only PA ↑ → ACM ↓ (−) PA ↑ → Recur. ↓ (x)
Phipps, A. I. (2018)	**[Purpose]** A study on the relationship between survival and recurrence according to physical activity patterns using clinical trial data of adjuvant chemotherapy for stage III colon cancer **[Setting]** The United States, multicenter **[Design]** Prospective, cohort study	**[Data]** The North Central Cancer Treatment Group (NCCTG; now a part of the Alliance for Clinical Trials in Oncology) Multicenter phase III randomized trial **[Duration]** DFS: f/u 5 yrs OS: f/u 8 yrs Until 2014.12.3 **[Cohort characteristics]** Total (stage 3) = 1992 Deaths = 505 Recurrence = 541	[Physical activity] * During a routine day: almost none/mild activity/moderate/heavy activity * Free time: - Never: about once a month/several times a month/several times a week/daily) - Moderate physical activity: golf, garden management, long walking, bowling - vigorous physical activity: jogging, racket sports, swimming, aerobics	**[PA−Overall survival]** Any free-time PA (+) PA > once/month (vs. ≤ once) [HR: 0.76 (0.63–0.93)] (vs. none) [HR: 0.73] Moderate-intensity ≥ 2/month (vs. ≤ once) [HR: 0.80 (0.66–0.96)] **[PA−Disease-free survival]** Any free-time (+) PA > once/month (vs. ≤ once) [HR: 0.82 (0.69–0.99)] (vs. none) [HR: 0.77] → Vigorous-intensity PA (−)	PA measurement after Dx (+) → before Tx PA ↑ → DSM ↓ (+) PA ↑ → ACM ↓ (+) PA ↑ Recur. ↓ (+) The prognostic effect of physical activity was better in non-smokers, T3, folfox monotherapy, and obese groups.
Van Blarigan, E. L. (2018)	**[Purpose]** Confirming whether following ACS guidelines improves the survival rate of colon cancer patients **[Setting]** The United States **[Design]** Prospective, cohort study	**[Data]** CALGB (Cancer and Leukemia Group B) 89803 Enrollment: 1999–2001 within 8 min after surgery. **[Cohort characteristics]** Enrolment: N = 992 Stage 3 colon cancer Recurrence = 335 Deaths = 299 Recurrence + deaths = 256/335 (86%)	**[Physical activity]** During and six months after chemotherapy MET: < 8.75; 8.75–17.4; ≥ 17.5 The average value is used after the survey on the 90th and 180th days	[PA−Overall survival] * 8.75–17.4 MET h/wk (vs. < 8.75) [HR: 0.64 (0.45–0.92)] : Median MET = 12.0 * ≥ 17.5 MET-h/wk (vs. < 8.75) [HR: 0.58 (0.42–0.81)]: Median MET = 32.2	PA measurement after Dx (+) PA ↑ → DSM ↓ (−) PA ↑ → ACM ↓ (+) PA ↑ → Recur. ↓ (−) * Recommendation at least 8.75 MET h/wk 8.75 MET h/wk = moderate activity (brisk walking) 150 min/wk
Kuiper, J. G. (2012)	**[Purpose]** The effect of recreation and physical activity on mortality before and after diagnosis in female CRC patients **[Setting]** 40 centers in the United States **[Design]** Prospective, cohort study	**[Data]** The Women’s Health Initiative study (WHI) October 1993–December 1998 40 centers in the United States **[Cohort characteristics]** Enrollment = 1339 women	[Physical activity] * MET - Mild (3 MET): slow dancing, bowling, golf - Moderate (4 MET): biking, exercise machines, calisthenics, easy swimming, dancing - Strenuous (7 MET): aerobics, jogging, tennis, swimming laps - MET Level (MET h/wk) 0.0; > 0.0–2.9; 3.0–8.9; 9.0–17.; ≥ 18.0	[PA−ACM] * Pre-diagnostic PA level ≥ 18.0 MET h/wk (vs. 0.0) [HR: 0.63 (0.42–0.96), *p* trend = 0.02] * Post-diagnostic PA level ≥ 18.0 MET h/wk (vs. 0.0) [HR: 0.41 (0.21–0.81); *p* trend = 0.005] [PA−DSM] * Pre-diagnostic PA level → 59% ↓ ≥ 18.0 MET h/wk (vs. 0.0) [HR: 0.68 (0.41–1.13), *p* trend = 0.08] * Post-diagnostic PA level → 71%↓ ≥ 18.0 MET h/wk (vs. 0.0) [HR: 0.29 (0.11–0.77); *p* trend = 0.02]	PA measurement before and after Dx (+) PA → DSM ↓ (+) PA → ACM ↓ (+) PA Recur ↓ (x) ≥ 9 MET h/week 32% ↓ DSM 37% ↓ ASM = moderate * 3 h/wk
Hardikar, S. (2015)	**[Purpose]** The effect of physical activity level before diagnosis on survival [Setting] Six study centers Seattle, United States **[Design]** Prospective, C ohort study	**[Data]** The population-based Seattle Colon Cancer Family Registry (S-CCFR) **[Duration]** f/u until December 2012 **[Cohort characteristics]** CRC pt. = 2706 not metastasis (excluding stage 4)	**[Physical activity]** - MET (+) MET h/wk < 3.5; 3.5 ≦ 8.75; 8.75 ≦ 17.5; 17.5 ≦ 35; ≧ 35 Cutoff—8.75 MET h/wk Moderate = 2.5 h Vigorous = 75 min	**[PA−OS]** < 3.5, Reference 3.5 ≦ 8.75, HR: 0.53 (0.39–0.72) 8.75 ≦ 17.5, HR: 0.64 (0.48–0.85) 17.5 ≦ 35, HR: 0.64 (0.47–0.85) ≧ 35, HR: 0.70 (0.52–0.96) **[PA−DSS]** < 3.5, Reference 3.5 ≦ 8.75, HR: 0.58 (0.39–0.86) 8.75 ≦ 17.5, HR: 0.56 (0.38–0.83) 17.5 ≦ 35, HR: 0.60 (0.40–0.88) ≧ 35, HR: 0.63 (0.42–0.95)	PA measurement before Dx (+) PA → DSM ↓ (+) PA → ACM ↓ (+) PA Recur. ↓ (x) PA beneficial effect - all molecular phenotypes of CRC (+)

PA: Physical activity; Dx: diagnosis; CRC: colorectal cancer; DSM: disease-specific mortality; ACM: all-cause mortality; MET: metabolic equivalent task; HR: hazard ratio; N/S: not significant; 5YS: five-year survival; DSS: disease-specific survival; AJCC: American Joint Committee on Cancer; DFS: disease-free survival; CPS I: the American Cancer Society Cancer Prevention Study II; ACS guidelines: The American Cancer Society Nutrition and Physical Activity Guidelines for Cancer Survivors; (x): not measuring; (+): relevance confirmed; (−): not relevant; NHS: National Health Service.

## Data Availability

Data sharing not applicable.
